# Odonata (dragonflies and damselflies) as a bridge between ecology and evolutionary genomics

**DOI:** 10.1186/s12983-016-0176-7

**Published:** 2016-10-10

**Authors:** Seth Bybee, Alex Córdoba-Aguilar, M. Catherine Duryea, Ryo Futahashi, Bengt Hansson, M. Olalla Lorenzo-Carballa, Ruud Schilder, Robby Stoks, Anton Suvorov, Erik I. Svensson, Janne Swaegers, Yuma Takahashi, Phillip C. Watts, Maren Wellenreuther

**Affiliations:** 1 Brigham Young University, Provo, UT 84606 USA; 2 Departmento de Ecología Evolutiva, Instituto de Ecología, Universidad Nacional Autónoma de México, Apdo, Postal 70-275, Ciudad Universitaria, 04510 Mexico City, Mexico; 3 Evolutionary Ecology Unit, Department of Biology, Lund University, 223 62 Lund, Sweden; 4 Bioproduction Research Institute, National Institute of Advanced Industrial Science and Technology, Central 6, Tsukuba, Ibaraki 305-8566 Japan; 5 Institute of Integrative Biology, Biosciences Building, University of Liverpool, Crown Street, Liverpool, L69 7ZB UK; 6 Departments of Entomology and Biology, Pennsylvania State University, University Park, PA 16802 USA; 7 Laboratory of Aquatic Ecology, Evolution and Conservation, Department of Biology, University of Leuven, 3000 Leuven, Belgium; 8 Department of Biology, Brigham Young University, LSB 4102, Provo, UT 84602 USA; 9 Division of Ecology and Evolutionary Biology, Graduate School of Life Sciences, Tohoku University, 6-3, Aoba, Aramaki, Aoba, Sendai, Miyagi 980-8578 Japan; 10 Department of Ecology, University of Oulu, Oulu, 90014 Finland; 11 Plant and Food Research Limited, Nelson, 7010 New Zealand

**Keywords:** NGS, Ancient insects, Complex life cycle, Naiad, Climate change, Polymorphism, Flight, Ecological Genomics

## Abstract

Odonata (dragonflies and damselflies) present an unparalleled insect model to integrate evolutionary genomics with ecology for the study of insect evolution. Key features of Odonata include their ancient phylogenetic position, extensive phenotypic and ecological diversity, several unique evolutionary innovations, ease of study in the wild and usefulness as bioindicators for freshwater ecosystems worldwide. In this review, we synthesize studies on the evolution, ecology and physiology of odonates, highlighting those areas where the integration of ecology with genomics would yield significant insights into the evolutionary processes that would not be gained easily by working on other animal groups. We argue that the unique features of this group combined with their complex life cycle, flight behaviour, diversity in ecological niches and their sensitivity to anthropogenic change make odonates a promising and fruitful taxon for genomics focused research. Future areas of research that deserve increased attention are also briefly outlined.

## Background

With more than 1,000,000 species described and an estimated 5,000,000 extant species, insects represent the most diverse animal taxon on Earth [1, 2]. They inhabit key roles as herbivores, pollinators, seed dispersers, predators, detritivores and vectors, thereby contributing to the core biological foundation of all terrestrial ecosystems [3, 4]. Insects are also of exceptional economic importance as providers of essential ecosystem services (e.g. global economic value of US$153 billion of insect pollination in 2005, [5]), pests in agricultural landscapes (e.g. annual control of the diamondback moth Plutella xylostella costs US$4–5 billion, [6]) and as vectors of diseases affecting humans (e.g. malaria control costs ~ US$12 billion annually, Centre for Disease Control).

Dragonflies and damselflies (Insecta: Odonata) represent a species rich, yet tractable (~6000 described species, [7]) insect order, which encompasses two main suborders, Anisoptera (dragonflies) and Zygoptera (damselflies). The former are generally larger and alight with their wings held out to the sides, while damselflies have slender bodies, and generally hold their wings over the abdomen when at rest. Here we will use the term odonate as the inclusive terms when referring to both suborders. Several characteristics make odonates an attractive group to combine ecology with evolutionary genomics. First, they are direct descendants of one of the most ancient winged insect groups and, along with Ephemeroptera (mayflies), are sister to all other neopteran insects [8]. Second, odonates incorporate rich phenotypic and ecological diversity in one single insect order and therefore constitute excellent candidates for ecological and evolutionary studies [9]. As such, they have been used extensively as model species in many areas of ecology and evolution, such as sexual selection, behaviour, evolution of flight and life history theory [9]. Third, the group shows several evolutionary innovations, particularly with regard to flight (e.g. direct flight musculature), vision (e.g. complex colour vision,) sexual behaviour (e.g. secondary genitalia), and life history (e.g. complex life cycle). Fourth, the large interspecific variation in habitat specificity and complex aquatic/terrestrial life cycles makes odonates prominent surrogates for evaluating all types of freshwater ecosystems worldwide [10]. Lastly, dragonflies and damselflies are comparatively large insects, both as adults and late-instar larvae, and as such their behaviours can be studied readily in the wild. Thus, the phylogenetic position of odonates, combined with their numerous evolutionary innovations make them an attractive model to bridge ecology with contemporary evolutionary genomics and can provide fundamental insights into the origin of these traits. Despite the attractiveness of this group for evolutionary genomics studies, efforts have been lagging behind other insect orders (see Table 1 for a summary of current genomic resources for Odonata).

**Table 1:** Genomic resources currently available for Odonata (as from 1^st^ of May 2016)

Type of resource	Suborder	Family	Species	Reference/s
Genomes	Anisoptera	Libellulidae	*Ladona (Libellula) fulva*	Available in GenBank,
				Bioproject PRJNA194433
ESTs	Zygoptera	Coenagrionidae	*Ischnura elegans*	[213]
Transcriptomes	Anisoptera	Libellulidae	*Libellula vibrans*	Available in GenBank,
				Bioproject PRJNA258192
			*Libellula fulva*	Available in GenBank,
				Bioproject PRJNA275663
			*Pantala flavescens*	Available in GenBank,
				Bioproject PRJNA239794
			*Sympetrum frequens*	[141]
			*Orthetrum albistylum*	[141]
		Cordulegastridae	*Cordulegaster boltonii*	[8]
			*Anotogaster sieboldii*	[141]
		Corduliidae	*Somatochlora uchidai*	[141]
		Macromiidae	*Macromia amphigena*	[141]
		Petaluridae	*Tanypteryx pryeri*	[141]
		Gomphidae	*Asiagomphus melaenops*	[141]
		Aeshnidae	*Anax parthenope*	[141]
	Anisozygoptera	Epiophlebiidae	*Epiophlebia superstes*	[141]
	Zygoptera	Coenagrionidae	*Enallagma hagenii*	[214]
			*Coenagrion puella*	[59]
			*Ischnura elegans*	[162]
			*Ischnura asiatica*	[141]
			*Ischnura ramburii*	Available in GenBank, Bioproject PRJNA270761, [215]
			*Telebasis salva*	Available in GenBank, Bioproject PRJNA270761, [215]
		Calopterygidae	*Calopteryx splendens*	[8]
			*Mnais costalis*	[141]
		Lestidae	*Indolestes peregrinus*	[141]
Mitogenomes	Anisoptera	Libellulidae	*Orthetrum triangulare melania*	[216]
			*Hydrobasileus croceus*	[217]
			*Brachythemis contaminata*	[218]
		Corduliidae	*Cordulia aenea*	[219]
		Gomphidae	*Davidius lunatus*	[220]
			*Ictinogomphus* sp.	[217]
	Anisozygoptera	Epiophlebiidae	*Epiophlebia superstes*	[221]
	Zygoptera	Coenagrionidae	*Ischnura pumilio*	[222]
		Euphaeidae	*Euphaea formosa*	[70]
		Pseudolestidae	*Pseudolestes mirabilis*	Available in GenBank, (FJ606784)
		Calopterygidae	*Vestalis melania*	[223]
			*Atrocalopteryx atrata*	Available in GenBank, (KP233805)
			*Mnais costalis*	[224]
		Platycnemidae	*Platycnemis foliacea*	Available in GenBank, (KP233804)

At present, most genomic resources for arthropods are available for dipteran flies [e.g. the many *Drosophila* species, http://flybase.org/static_pages/species/sequenced_species.html], lepidopterans (e.g. moths and butterflies, [11]) and hymenopterans (e.g. wasps and bees, [12]). To some extent, this taxonomic bias is caused by the large economic and/or medical importance of these groups and in some cases because they serve as key laboratory models [13]. However, while it is true that model species provide numerous insights into key molecular and evolutionary processes, they do not necessarily capture essential parts of the biology and ecology of their relatives, especially in the case of those species that are more distantly related. By casting a small net for insect genomic resources, only a partial picture of insect adaptation is formed. Moreover, a focus on a few model organisms could promote a confirmation bias - a “tendency to see what we expect to see” [14]. Conversely, research into diverse taxa will contribute new knowledge to the current “omics” (e.g. genomics and transcriptomics) era. Such an approach offers insights into molecular adaptive processes that occur at contemporary and phylogenetic timescales and are of relevance to ecosystem functioning and stability [15–17].

Recent advances in high-throughput sequencing technologies make it possible to generate large amounts of sequence data from virtually any organism, at a rapid rate and at relatively low cost [18]. Thus, it is now possible to bridge the gap between ecology and genomics and to connect the often unique and well-studied evolutionary ecology of Odonata with a genomic perspective. This advancement will not only increase our ability to understand evolutionary processes within the group, but also add fundamental insights across insects. In this review, we outline the central research importance of Odonata by presenting some of the key features that make this group an unparalleled model system to integrate genomics with ecology and evolution. We synthesize the work conducted on the evolution, ecology and physiology of odonates, as well as the contemporary research showing that they are amenable study species to quantify responses to anthropogenic change, to inform conservation efforts. We focus specifically on those areas where the integration of genomics with ecology and evolution would yield significant insights into evolutionary dynamics that would not be easily gained by working on other animal groups. Finally, we outline future areas of research that deserve increased attention.

## Taxonomy and phylogenetic position

**Fig. 1:**
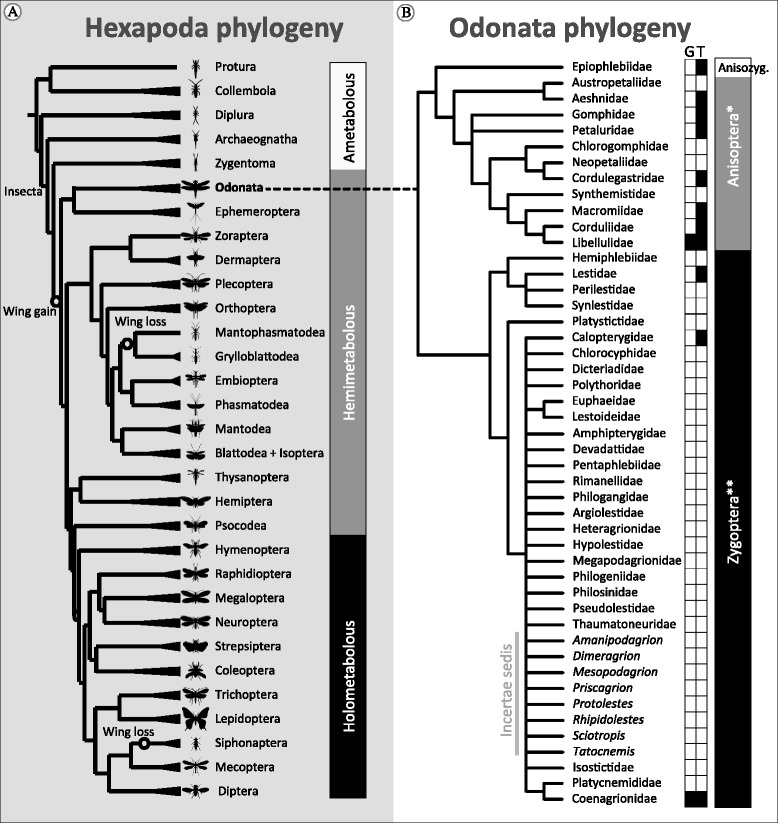
Phylogenetic position and phylogeny of Odonata. **a** Phylogeny of Insecta, showing the position of Odonata. Redrawn from Misof et al. [8] by R. Futahashi. **b** Recreation of Odonate phylogeny showing the current state of odonate phylogeny. Redrawn and synthesized from [23] and [20]. While a clear picture of family level groupings has emerged a well-supported backbone of phylogenetic relationships for both Anisoptera and Zygoptera is still lacking. G = (draft) genomes available and T = transcriptomes available (see Table 1 for details). Note that the two draft genomes currently available could not be more distantly related and there is a need to close the more than 250,000,000 year gap that exists between the two species

Dragonflies and damselflies (Fig. 1a, [8]) are extant representatives of the first ancient winged insects. Their phylogenetic position makes this group of central importance to comparative studies on the evolution of genomic innovations involved in the origins of physiological processes (e.g. flight, colour vision, and metabolism) and life history strategies (e.g. predation, mating, dispersal, and complex life cycles). Thus, a tractable and large-scale phylogeny would provide a rigorous framework to quantify evolutionary changes in genome architecture and provide insight on the origin of evolutionary innovations in odonates and insects in general.

Over the past 20 years, much progress towards reconstructing the phylogeny of Odonata has been made [8, 19–23]. Noteworthy, efforts to construct a detailed classification scheme based on the solid phylogenetic support of suborders and families, albeit some branches still show low support and conflict among families [19, 20, 24–26]. A key limitation of these studies is their reliance on a small set of loci for phylogenetic reconstruction [25, 27]. More robust results are likely to be found with genomic approaches for phylogenetic estimation [28, 29], but before these tools can be properly designed and utilized to quantify phylogenetic relationships within Odonata, genome-level data are necessary. One goal of the 1KITE project [8] is to produce transcriptomes of 107 odonate species (representing roughly half of the family-level diversity), which will provide the first “big data” estimates of the phylogeny of the group and address some higher-level problems of classification (e.g. relationships between the anisopteran families and corduliid monophyly). These data will provide important information to both systematists and ecologists and evolutionary biologists in general by providing a much-needed perspective to address general questions in odonate biology in a phylogenetic context. Future efforts to resolve the phylogeny by obtaining transcriptomic or genomic data for other odonate species will further help to broaden our perspective on this group and provide greater insight into their evolutionary biology.

With approximately 6000 species currently described [30], the taxonomy of the Odonata has been largely considered as well established, and it has been estimated that 95 % of all extant species will be described by 2030 [7]. However, less explored regions, like the Neotropics and the African continent, are likely to harbour a high number of species not yet known for science (e.g. see [31] for the recent description of 60 new dragonfly species). Taxonomy is a very important scientific field, and a correct identification of organisms constitutes an essential infrastructure for other research areas [7]. The numerous high-throughput technologies currently available allow for the characterization of the genome, transcriptome, proteome and even the morphology of an organism (e.g. CT scans, [32]). The application of such technologies to taxonomic research in dragonflies and damselflies would improve the quality and quantity of data that can be applied, not only to the description of new species, but also to provide new perspectives for the correct identification of specimens [33].

### Fossil record

**Fig. 2:**
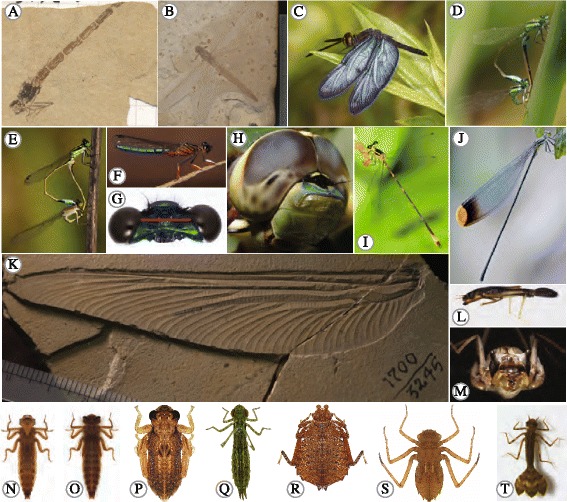
Diversity of both fossil and extant Odonata. **a** *Lestes ceresti* paratype MNHN R0744 Paris, France. Specimen is from the Chattian lacustrine carbonite of France and is 23.03–28.4 Ma. **b** *Isophlebia sp.* MNHN R55232 Paris, France. **c** *Zentihoptera lanei* **♂** courtesy of J. Johnson. **d** *Ischnura ramburii* mated pair (male above female below) with an andromorphic female, courtesy of S. Coleman. **e** *Ischnura ramburii* mated pair with a gynomorphic female courtesy of S. Coleman. **f** *Platycypha caligata* **♂** courtesy of J. Abbott. **g ** Head of *Calopteryx maculata* **♂** showing the general head shape and relationship of the eyes and antennae for damselflies (Zygoptera). *Red bar* shows the distance between the eyes. **h ** Head of *Anax junius* **♂** showing the general head shape and relationship of the eyes and antennae for dragonflies (Anisoptera) courtesy of R. Nelson. **i** *Heteragrion angustipenne* **♂** courtesy of K. Tennessen. **j** *Microstigma rotundatum* **♂** courtesy of K. Tennessen. **k** *Arctotypus sylvaensis* holotype PIN 17000/3245 Moscow, Russia. **l** *Philogenia mangsisa* larva from Bybee and Tennessen 2008. **m**-**o** *Cordulegaster* sp. larva anterior, dorsal and ventral view respectively. **p** *Epiophlebia laidlawi* larva. **q** *Anax junius* larva*.* **r** *Hagenious brevistylus* larva. **s ** Macromiidae sp. **t** *Podolestes orientalis* larva courtesy of C.Y. Choong*.* M-S from SMB

Odonates have one of the most complete and well-preserved fossil records among insects (Fig. 2). The Protodonata represent a fossil crown group to the extant Odonata and first appeared in the Namurian of the Carboniferous around 319,000,000 years ago). Protodonate fossils show evidence of many important traits that are still exhibited by extant odonates, such as an aquatic immature stage, a complex life cycle [34], and the complex mating system that typifies this group (i.e. males use secondary genitalia to transfer sperm, see also section 2E, [35]). The earliest fossils that are recognized as “modern” odonates date to ~268 Mya from the Upper Permian (*Saxonagrion minutus*, [36]) soon after which several stem group fossils for each of the modern suborders appeared, representing many of the families and even some modern genera. The extensive fossil record for several contemporary odonate groups, in combination with information of their relative ages from genomic data, offers the prospect of a thorough integration between the fossil record and contemporary studies of evolutionary biology that will help to shed significant light on many evolutionary questions. For example, insect flight is particularly intriguing as it likely evolved only once [37], and the anatomical regions from which wings evolved among the early insects are as yet unknown. A combination of genomic resources, fossils and evolutionary development approaches may help to identify the genetic toolkit responsible for insect flight, as well as the loci associated with key innovations during the evolution of wing morphology as different odonate species diversified and colonised new habitats.

### Genome size

The so-called “C-value enigma” refers to the observation that the genome size among many eukaryotes can vary widely, and this variation does not have to correlate with the number of genes or the organismal complexity (e.g. some unicellular organisms have genomes much larger than humans). Understanding how and why genomes show such pronounced size variation has become a timely research topic, especially in the current post-genomics era [38].

Much has been discovered about the patterns and consequences of variation in genome size, with most of these discoveries coming from studies on vertebrates and plants (e.g. [39, 40]), and comparatively little from insects. The first comprehensive study of genomic variation in odonates quantified genome variation in 100 North American species, and revealed a nearly ten-fold difference in genome size between species (from 0.41 pg (*Miathyria marcella*) to 2.36 pg (*Somatochlora elongata*), [41]). Genome size correlations with voltinism and larval habitat were not found, but a significant relationship between genome size and body size (positive in dragonflies and negative in damselflies), and flight ability was found (with small genomes being associated with percher species, that is those that only fly intermittently in between periods of perching, and large genomes with fliers, that is those that fly continuously). Finally, genome size was also positively correlated with a species’ chromosome number [41]. Future work using a combination of genomic and transcriptomic data could be used to elucidate putative mechanisms responsible for the variation in genome size across odonate species; such as gene duplication, DNA loss, variation in intron size or transposable elements.

## Evolutionary ecology

Modern odonates have an exceptionally well-documented behaviour and natural history [9]. The Holarctic regions have the best described odonate faunas, while the greatest species diversity and most understudied faunas are found in tropical areas. Keys and field guides for adult odonates are available for most areas of the world [42–45], and the techniques to observe and capture individuals can be learned with relative ease, making odonates one of the few insect groups with large and comprehensive insect collections (e.g. Florida State Collection of Arthropods, Naturalis Biodiversity Center in Leiden, The Netherlands). Characteristics such as their relatively large body size and conspicuous behaviour make them an ideal insect group to study components of adult fitness in natural populations [46–49]. Below we highlight the distinct ecological traits of odonates that make them a remarkable study system for connecting field ecology with general questions in biology, including the evolution of complex life cycles, fitness consequences of divergent reproductive modes and behaviours, response to climate change, and the evolution of flight.

### Complex life cycle

Most animal species (80 % of the animal kingdom) have a complex life cycle (CLC), whereby the immature and adult stages occupy different ecological niches and often undergo varied degrees of metamorphosis [50–52]. Odonata make an excellent group to explore the evolutionary causes and consequences of CLCs as the larvae are aquatic and the adults are terrestrial, and both life stages are well-studied [52]. Organisms that live in different environments throughout their ontogenies are faced with constraints to optimize responses to the various selection pressures that operate in each environment [53]. Thus, the relevant question concerns how one genome responds to contrasting selection regimes in multiple environments. Moreover, when these environments undergo divergent changes, for example through global warming which will affect aquatic and terrestrial habitats differently [51], one genome must mediate appropriate genetic responses in two different life stages across two different and changing environments. Studies of such genetic (including epigenetic) responses in odonates can be used to understand how other animals with CLCs may respond to climatic changes and in what systems adaptations are likely to occur. A major hurdle to the study of CLC evolution is a lack of knowledge about the extent to which life stages genetically covary and whether selection acts in a complementary (or divergent) way [54, 55]. The few quantitative genetic studies that have addressed this issue found support for genetic associations across life stages, but also showed that traits are capable of independent evolutionary change in response to the divergent conditions encountered during each life stage (ascidians [55], or anurans [56]). It thus seems that both genetic association and independent evolution can help to shape adaptation in some species, however, the paucity of studies to date make it impossible to draw general conclusions.

The molecular mechanisms underlying the coupling of life stages across metamorphosis are not particularly well studied (but see [57] for work on *Drosophila*). Thus, there is a major gap in understanding gene-by-environment interactions that would occur during major developmental transitions [58]; for example, how do the immune systems of dragonflies and damselflies respond to different larval and adult environments? The recently identified immune genes in the damselfly *Coenagrion puella* [59] would allow testing more directly whether larval and adult stages evolve independently from one another. Furthermore, transcriptomic studies measuring gene expression patterns during larval and adult stages would elucidate the degree of plasticity in gene expression in different life stages and how environmentally induced changes differentially affect the genetic responses of larval and adult life stages [58]. Such studies would improve our understanding of how differential selection pressures across the life cycle modulate genetic and plastic adaptive processes. A genomic approach would also provide an important complement to understanding the documented carry-over effects of larval stressors to adult fitness. For example, it has been demonstrated that larval food shortage affects adult lifetime reproductive success in the damselfly *Lestes viridis* [60]. Moreover, transcriptomic studies could address the extent to which epigenetic changes in the larval stage are reprogrammed during metamorphosis [61], which may facilitate novel epigenetic responses at the adult stage.

### Movement dynamics: dispersal

Dispersal is a fundamental ecological and evolutionary process that redistributes individuals among areas [62], thereby buffering against the demographic and genetic losses that are expected to occur in otherwise isolated populations. Perhaps the best-studied animal in terms of dispersal ecology and concomitant eco-evolutionary dynamics is the Glanville fritillary butterfly *Melitaea cinxia* [63]. This was one of the first non-laboratory organisms studied using high-throughput sequencing approaches [64], which demonstrates not only the feasibility but also the usefulness of obtaining genomic data from wild populations. Work on the Glanville fritillary quantified how polymorphisms at a single locus can be associated with population demography [65] and life history traits and fitness in both adults and larvae [63, 66, 67]. Odonates share many of the attractive features of butterflies for integrative research into movement dynamics, including the ease to study larval life history traits, and adults that can be marked and recaptured to quantify dispersal and mortality in the wild. In addition, odonates provide the added dimension of linking terrestrial and aquatic systems.

Dispersal can be quantified using both ecological and genetic methods. Indeed, studies on odonates have provided evidence that such different methodologies provide comparable information about population connectivity [68, 69]. Odonates have provided model systems for studies of how landscape features, such as urban areas [68] or high grounds [70], can limit dispersal and how agricultural development may affect dispersal pathways [71]. These studies also uncovered a loss of genetic diversity in isolated populations [72], but there is little information about the eco-evolutionary consequences of genetic erosion in odonate populations. The application of genomic techniques to quantify, for example, whether, and if so how, small population size limits adaptation in wild populations would be useful for informing conservation management.

### Monitoring the consequences of climate change

Several damselfly species have modified their distributions and abundances over the last few decades in response to rising global temperatures [73–75]. Long-term distributional data of adults show that odonates are amongst the taxa showing the strongest poleward range expansions [73, 74], making them excellent study organisms for unravelling the still poorly documented rapid microevolutionary changes associated with range expansions [76]. This research can be embedded in the several well-documented cases of latitudinal adaptation among odonates. For example, common garden studies on larvae of the damselflies *Ischnura elegans* and *Lestes sponsa* provided a detailed picture of thermal adaptation along a latitudinal gradient in Europe. A key pattern is the evolution of thermal reaction norms and voltinism in response to differences in temperature [77]. Notably, the evolution of higher thermal optima and faster growth rates in southern latitudes has been associated with changes in digestive physiology [78], cold resistance [79], predator–prey interactions [80] and resistance against contaminants [81]. Other studies in *L. sponsa* indicated the evolution of larval growth and development rates and their response to photoperiod [82–84]. Genomic studies for these cases of latitude-associated adaptation may not only reveal the pathways underlying the observed phenotypic differentiation but may also identify novel aspects of adaptation along this strong thermal axis. Variation in these candidate genes can then be screened in spatial and temporal contexts as climate change continues.

Recent work aimed to quantify the genetic consequences for odonate species that are expanding their ranges has shown reductions in genetic diversity in edge-of-range populations [85, 86], and evidence for selection at the gene level [87]. Using common garden experiments, rapid evolution of both larval traits (increased growth rate and increased activity levels) and adult traits (increased flight ability and increased immune function) was demonstrated in the rapidly poleward expanding damselfly *Coenagrion scitulum* [88, 89]. The few studies on genomic signatures of range expansion in both plants and animals did not link genetic changes to phenotypes and did not unravel the evolutionary processes involved [76]. In a first effort to do so, a single-nucleotide polymorphism (SNP) study focused on *C. scitulum* revealed one SNP associated with increased flight performance to be under consistent selection in the populations at the expanding range edge [87]. This indicates that evolutionary changes among independent edge populations are driven by the range expansion process *per se*. This study illustrates the added value of integrating genomic, phenotypic and environmental data to identify and disentangle the neutral and adaptive processes that are simultaneously operating during range expansions. An important future step will be to identify other determinants of dispersal ability at the molecular level. For example, some sequence data on candidate ‘dispersal’ genes, such as *pgi*, *hif1alpha* or *sdhd* [90, 91] are available for odonates [27]. Applying genomic studies to the other well-documented range expansions in odonates may therefore considerably add to the limited knowledge on how species evolve during range expansions.

### Climate change and hybridisation

In addition to a loss of species diversity, many range expansions are creating *de novo* sympatric areas between formerly allopatric taxa, and increasing evidence is suggesting that this can modify species interactions [92]. Furthermore, evidence is growing that species interactions in these newly created sympatric zones are leading to the breakdown of species barriers and rapid hybridisation (reviewed in [93]). Thus, species identities in these *de novo* sympatric zones may be unclear. Molecular methods for species identification could help us to resolve species identities via genetic means and provide clues about the general processes underlying the creation of biodiversity. For example, by studying the genomics of species hybridisation and species introgression in odonates, we would obtain a better knowledge of the processes underlying the creation of novel genetic adaptations. In general, it is thought that adaptive genes have a greater chance to cross species boundaries than key “speciation genes” or genes residing inside “genomics islands of divergence”, which should both be more resistant to introgression [94, 95]. Genomic studies on introgressive hybridisation in damseflies are being initiated and have the potential to uncover if certain genomic regions are repeatedly inherited from the same parental species. These studies may be able to elucidate the size of genomic linkage islands and how the inheritance of genomic regions correlates with morphology and ecology.

The vulnerability of odonates to anthropogenic changes makes informed conservation measures a priority, given the likely impact that these changes may have on the overall species diversity, food web structure and ecosystem stability. A recent comparative study on several damselfly species assessed the potential to use quantitative predictions of reproductive isolation as an indicator to assess species’ hybridisation risk [96]. The study found a positive correlation between the degree of reproductive isolation and genetic distance between species, as has been shown in fruit flies [97] and butterflies [98]. This clear link between species divergence rates and the likelihood to hybridise strongly suggests that genetic divergence between taxa can be used as a proxy to predict hybridisation rates of species that come into contact following climate induced range expansions [96]. This link can be used to inform conservation efforts, particularly for odonate species that are already endangered (e.g. *Ischnura gemina*, [96]).

## Sexual selection

**Fig. 3:**
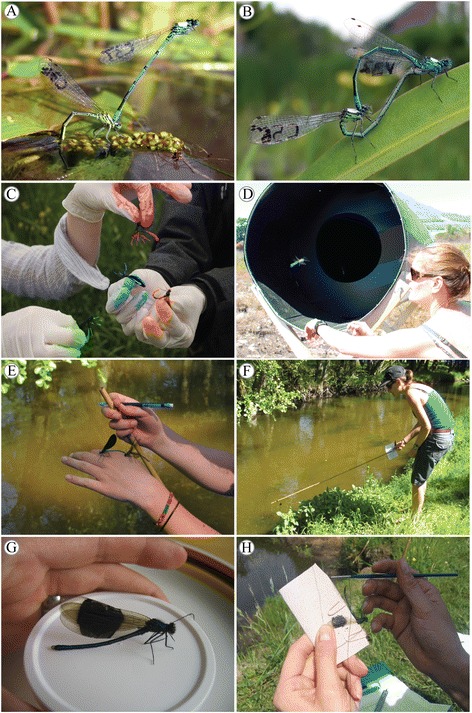
Some field applications using Odonata. Panel **a**-**b** show pairs of the damselfly *Coenagrion puella* at Queen Elizabeth Country Park, UK. Animals have been marked on the thorax (to easily identify marked from unmarked animals) and assigned a unique code on the hindwing so that individual behaviours at the mating site can be recorded throughout an entire breeding season (photo credit Phillip C. Watts). Panel **c** shows males of the damselfly *Calopteryx splendens* (different colours represent groupings of resident and immigrant males, as well as mature and immature males) that have been marked with fluorescent dye at Klingavälsåns Naturreservat in Sweden to be measured upon release with a LIDAR [225]. Panel **d** shows an unidentified anisopteran species that was released at Stellenbosch in South Africa for trialling the setup of a remote insect monitoring technique called dark field spectroscopy [226]. Panel **e** shows a *Calopteryx virgo* damselfly male interacting with female *C. virgo* at Sövdemölla in Sweden. The female has been tethered with a cotton string to a bamboo stick to record mating responses of males. Panel **f** shows how the same tethered female from Panel **e** is being moved along the stream shoreline to record male responses [227]. Panel **g** shows a *Calopteryx splendens* male that had his wing patches increased with black paint, and Panel **h** shows how such wing manipulation can be applied even under field conditions. Photo credits C-H Maren Wellenreuther

Odonates are key players in the understanding of sexual selection theory and have been traditionally used as models in studies of sexual conflict [99], character displacement [100, 101] and sexual selection in relation to colour polymorphism and sperm competition [102]. Their fidelity to reproductive areas (particularly males), diverse reproductive behaviour and amenability to phenotypic manipulation make them exemplary systems for field studies (Fig. 3), behavioural observations, and laboratory experiments [103]. Indeed, few animal groups can rival Odonata for the combination of these traits (perhaps only water striders [104]).

Below, we highlight how genomic tools can be used to increase our understanding of the underlying evolutionary processes of sexual selection. Specifically, we focus on 1) evolution and origin of a unique reproductive mode, 2) genetics of mating behaviour and 3) how odonates are models for studying sexual dimorphism and sex-limited polymorphisms.

### Reproductive mode and behaviour

Insects are incredibly diverse in their reproductive behaviour and genetic tools are beginning to shed light on how the different reproductive modes have originated. To date, our knowledge of the genetics of insect reproductive behaviour comes mainly from studies of laboratory model species like *Drosophila* (e.g. [105]) and eusocial insects (e.g. [106]). Dragonflies and damselflies have a unique mode of reproduction whereby the male grasps the female by the head (dragonflies) or the prothorax (damseflies) and then the female raises the tip of her abdomen forward to receive sperm from the male secondary genitalia; forming a characteristic ‘mating wheel’ (Fig. 3b in [107]). Elucidating the genes involved in courtship and mating among odonates will help to clarify the evolutionary origin of their unique reproductive mode. Additionally, because modern odonates represent some of the most ancient insects, by identifying the genes involved in mating in this group, we can make evolutionary comparisons of the origins of reproductive behaviours in other well-studied insect groups.

Odonates are also a model system for studying sperm precedence. Males engage in various strategies to ensure reproductive success by removing or displacing rivals’ sperm from the female storage organs before transferring their own sperm [108]. Further studies of these behaviours using genomic tools can give us insight on the evolutionary origins of these diverse reproductive mechanisms and the large variation in female and male mating rate that promoted their evolution. In *Drosophila*, selection for increased mating rate led to major genetic changes (up to a 21 % of the entire genome) which pleiotropically selected for key functions related to neurogenesis, metabolism, development and general cellular processes [109]. In Odonata, genomic studies could address whether such disparate mating behaviours have also selected for other key biological functions, which may explain the extensive variation in ecologies (e.g. adaptation to tropical and non-tropical environments) observed in many closely-related species. To this extent, ischnuran damselflies may give unique insight because they occupy a variety of extreme environments and exhibit also a vast variation in mating strategies, ranging from polyandry to parthenogenesis. The latter is particularly interesting, because in contrast to other known insect groups in which asexual reproduction is frequently found, only one case of obligate parthenogenesis is known within the Odonata (the only-female populations of the American species Ischnura hastata on the Azores islands [110]). Ongoing comparative transcriptomic studies on sexual and parthenogenetic lineages of this species will help to better understand which genes are related to asexual reproduction and why it has evolved in this but not other species of this group.

With regards to the intra- and interspecies variation in sperm displacement mechanisms, nothing is yet known about the genetic underpinnings. Genomic studies that reveal these underpinnings can provide answers to several questions. Firstly, is sexual selection on genitalic function involved in population divergence and speciation [111]? Candidate genes for addressing this question are available from genetic work on male genitalic structures [112, 113] and female sperm storage organs in *Drosophila* [109] and eusocial insects. In particular, odonates exhibit extensive intra- [114] and inter-specific [115] variation in the morphology of female sperm storage organs. Secondly, do females gain indirect (genetic) benefits from mating multiple times [116] (e.g., through the production of more genetically variable offspring)? And thirdly, how can sperm remain viable once stored in the female sperm storage organs? For example, *Ischnura aurora* mate soon after emergence and then disperse, which implies female adaptations to keep sperm viable even when the animal is not sexually mature [117].

Dragonflies and damselflies also exhibit diverse pre-mating behaviours related to male-male competition. For example, distinct behavioural differences exist between territorial and non-territorial males, both within and among species [118]. Although ultimate effectors and fitness trade-offs of male mating tactics are reasonably well-known [119], knowledge of both the underlying genomic basis and hormonal influences are lacking [120]. There is potential for strong pleiotropic effects in some species, as seen in Japanese *Mnais* damselflies, where male mating tactics are linked to a male-limited colour polymorphism [121]. In this case we know that the expression of territorial behaviour is correlated with levels of juvenile hormone [122, 123], lipid content [124], muscular activity [125], infection level, and flight muscle protein expression [126]. Studies indicate that these pathways are highly conserved likely due to purifying selection [127], signifying that the widespread variation in male odonate sexual behaviour may be driven by mutations in gene expression profiles rather than changes in protein coding sequences.

Odonata has also been an exemplary group for studies on female preference. Several damselfly species appear to exhibit learned mate behaviours and plastic mate preferences [128], and populations commonly show pronounced preferences even across small spatial scales. The extent to which population divergence is related to mating preference is relatively unexplored, but it is likely that the combined action of learning, plasticity and microevolutionary processes are involved in most cases. For example, it is known that naïve female *Calopteryx splendens* can rapidly learn to distinguish between con- and heterospecific males based on their wing phenotype [129, 130], and it appears that learning of heterospecific phenotypes may also be involved in sexual isolation between the European *Calopteryx* species [92]. In the latter study, it was shown that *C. virgo* males have lost part of their mate recognition ability and that this loss increased heterospecific mating attempts [92]. While association learning is probably partly involved in the increased heterospecific mating rates in allopatry, loss of mate discrimination alleles as a result of selection (e.g. reinforcement) or genetic drift in this case also likely have played a role. By combining behavioural field data with genomic data, we could determine to what extent mating preferences and species recognition are fixed at emergence, and to what degree populations with divergent sexual preferences differ in their genomic signatures. A combination of gene expression studies and mating trials would provide yet another way to gain deeper insights into the involvement of microevolutionary processes in sexual divergence in this group.

### Sexual dimorphism

#### Body size

Most odonate species show sexual dimorphism in colour and/or size (Fig. 2). Of these, sexual size dimorphism (SSD, a difference in body size between males and females), is often the most conspicuous feature between the sexes. SSD in adult odonates can emerge even when larvae are initially monomorphic [131]. Both types of SSD occur in Odonata; males of some species are larger than females (e.g. Calopterygidae) while females are larger than males in other species (e.g. Cordulegastridae) [132]. Inter- and intrasexual selection has been shown to facilitate male-biased SSD (i.e. larger males), particularly in species with territorial males [118, 132, 133]. However, when SSD occurs in odonates, then female-biased SSD (i.e. larger females) is often the rule. One explanation for this female-biased SSD is that selection for manoeuvrability during aerial encounters may select for reduced male size [134]. In concordance with this, male-biased SSD is common in non-territorial odonate species [132], where males rarely engage in aerial contests [107]. Interestingly, damselflies are more likely to exhibit SSD than anisopterans [118], although the reasons for this are still unclear. Female-biased SSD can also evolve as a consequence of fecundity selection [47, 118, 135].

SSD in odonates has only been explored in terms of sexual selection and mating systems, but exceptionally little is known about the underlying genetic basis of SSD in this group. Several gene candidates exist that may aid our understanding of the genetics of SSD, e.g. juvenile hormone and insulin. Juvenile hormone is a gene with highly pleiotropic functions, and among other things, is known to be related to body size [136] and the reproductive behaviour in several other insect species [137]. Thus by clarifying the role of juvenile hormone in producing and regulating juvenile hormone levels in odonates may provide the first pieces to understand not only the evolution of SSD, but also mating tactics which are frequently influenced by body size [138]. For example, it would be interesting to see how genes that regulate hormonal levels act pleiotropically by regulating energetic resources (e.g. lipid reserves) when switching mating tactics.

#### Colour

Sexual dimorphism in colour is also prevalent in Odonata, particularly in damselflies (Fig. 2), where intra-and interspecific interactions are commonly based on both body and wing colour phenotypes [139–141]. Many species-rich families live in open areas (e.g. ponds, marshes and streams) where body colour patterns could function as a trait for species recognition and intraspecific communication [142]. For example, conspicuous male colour evolution is commonly explained to result from female mate choice (i.e. intersexual selection) and/or male-male competition. In some cases, male wing colouration communicates the bearer’s condition to male and female conspecifics, as documented by an association between wing colouration and lipidic muscular content which is important for flight ability [143]. Further, males of some species show nuptial colour changes via chemical reduction of epidermal pigments [144]. Interestingly, evidence suggests that female wing colour in some calopterygid species evolved as a correlated response to selection on male wing colour, and was subsequently lost via natural selection [145]. Still, in females of some calopterygid species, wing colour is known to signal fecundity to males, presumably to provide females with the benefit of reduced male mating harassment through guarding after mating [146]. Thus, colouration can be related to male as well as female mate choice in odonates.

#### Colour-polymorphism and its origins

Cryptic female colouration in odonates is thought to have evolved to avoid excessive sexual male mating harassment [147], which is also thought to be related to the high frequency of female-limited colour polymorphism in this group [148]. Female-limited polymorphisms are often characterized by an andromorph and one or more gynomorph females. Andromorphs look and often behave like males, whereas gynomorphs exhibit a more cryptic and female-like colouration [149]. While sexual conflict over mating rates has been implicated as the main evolutionary force maintaining these polymorphisms, frequency-dependent mate choice is an additional process contributing to its maintenance, since rare morphs experience reduced harassment, thus creating cyclic dynamics in morph prevalence [150]. A recent study showed that population fitness is related to the frequency of female morphs, with the population overall experiencing higher fecundity when morph population frequencies are balanced [151]. Morph frequencies can vary across environmental gradients. For example, a latitudinal cline in andromorph morph frequency in *Ischnura senegalensis* is an apparent classic signature of natural selection acting on colour, with the fitness of andromorphs increasing with latitude implying a gene-by-environment interaction [152]. In contrast, morph frequencies of *I. elegans* in Sweden were stable over ten generations, consistent with the action of negative frequency-dependent selection [153]. A better understanding of species differences in colour gene frequencies across environmental gradients and populations will likely come from studies investigating the genomic architecture of colour in related species and would help us to address how sexual selection and sexual conflict operate not only in odonates, but also in other systems showing sex-limited polymorphisms [148].

#### Genetic architecture of colour polymorphism

Breeding experiments to understand the genetic basis of female colour in polymorphic damselfly species have shown that colour inheritance is consistent with a classic Mendelian pattern, involving a few alleles at a single locus [154, 155]. Recent and more detailed genomic work on several colour polymorphic butterfly species has shown that colour is commonly controlled by supergenes [156–158], which prevents the fine mapping of genes due to strong linkage [159]. A supergene architecture of tightly linked genes seems likely to be involved in some damselfly species as well, where multiple phenotypic and fitness-related traits correlate with colour [160], resulting in integrated yet discrete multivariate colour phenotypes. One of these species is *I. elegans*, where colour is controlled by three alleles in a dominance hierarchy [155], and affects several additional fitness related traits (e.g. development time, fecundity and egg morphology, [150, 161]).

Chauhan and co-workers [162] analysed the transcriptome of *I. elegans* and validated the presence of 12 genes in three pigmentation pathways, namely the pteridine, melanin and ommochrome pathway, thereby providing a good transcriptomic resource for future work on colour polymorphisms. Still, we know little about the candidate loci that govern colour in this and other odonate species, and much additional work is needed to determine the functional significance of pigment genes. Studies have been initiated to map the genomic regions underlying colour in *I. elegans*, through the generation of a linkage map [163], transcriptome assembly [162] and a draft genome (Wellenreuther et al. unpublished data). Once the genomic location of colour has been described in this species, the fitness effects in males and larvae that carry different colour genotypes could be evaluated (colour is only visually expressed in mature females and thus invisible in males and larvae). Likewise, by knowing the genomics of colour one could study the evolution of the colour polymorphism in families in which polymorphisms frequently occur. A clear candidate for such a study would be the family Coenagrionidae, which has >90 polymorphic species in the Holarctic alone [164].

An examination of how colour has evolved through the identification of not only the genes but also the pathways responsible for the diversity and maintenance of colour would have broad ramifications for our understanding of colour signalling (i.e. how colour may impact both the behaviour and overall ecology) among insects, and across the animal kingdom as well. For example, population genomic studies and gene network analyses across species could clarify the evolutionary origins of life history traits correlated with body colour, including morphology, reproductive traits and mating behaviour. These investigations, in combination with studies of the genetics of mating behaviour, have the potential to elucidate some of the mechanisms that underlie the widespread sexual conflict in this group.

## Physiology

There is a paucity of understanding of how genome sequence variation affects physiological mechanisms responsible for trait variation and evolution in odonates. In part, this reflects the non-trivial nature (e.g. rearing time and laboratory infrastructure investment) of maintaining larvae and adults in the laboratory, and of developing appropriate molecular tools (e.g. antibodies, PCR primers) that are needed to examine mechanistic features of trait expression at a physiological level. The availability of genomic resources for odonates would lower some of these technical thresholds and create opportunities to enhance the molecular-level understanding of well-studied traits, as well as ignite interest in novel fundamental and comparative physiological research on this insect group. Below we highlight several key physiological research themes for which the availability of genomic resources would significantly facilitate mechanistic understanding.

### Flight

From the moment of emergence from the aquatic environment, odonate individual fitness is critically dependent on an individual’s ability to fly. Species exhibit wide (~100-fold) variations in body mass and thus have evolved different flight behaviours and motor designs to accommodate this morphological diversity [165]. This variation in flight adaptations makes them excellent models to examine the genomic architecture associated with variation in flight capacity. For example, percher and flier dragonfly species differ in diurnal activity pattern and flight thermoregulatory strategy [166]. Moreover, odonates exhibit distinctly different flight system morphology and kinematics (e.g. wing beat frequency and amplitude [167, 168]). Indeed, maximum lift production per unit mass of the damselfly flight motor is significantly higher (~86 N/Kg) than that of dragonflies due to the lift enhancing “clap and fling” mechanism that they employ in flight (~54–60 N/Kg, [169]). Similarly, flight motor investment during sexual maturation varies dramatically among odonates, with males of territorial species showing relatively high flight muscle mass accretion [165], thus enhancing their flight-muscle ratio and aerial manoeuvrability. It is the continuum of odonate life history strategies that rely largely on flight that makes them such an attractive group for examining the mechanisms that operate to optimize flight motor design [126], neural control [170], comparative biomechanics [171] and closely associated features (e.g. vision, thermoregulation), across different ecological niches and in response to environmental variation. Interestingly, odonates have one of the few extant direct synchronous locomotor flight systems in the insect world, making them more similar to vertebrate musculoskeletal systems than many other insect groups.

An example of integrative work on molecular mechanisms controlling odonate flight performance is that on quantitative alternative splicing of the gene encoding troponin T, a key flight muscle sarcomere protein, in *Libellula pulchella* (reviewed in [172]). This gene regulatory mechanism appears to serve as a key controller of energy consumption and flight muscle performance throughout adult life [125]. It is sensitive to nutrition and body weight variation and impaired by an infectious agent from the environment, which causes a metabolic syndrome and impaired locomotion similar to that occurring in mammalian obesity [173]. The potential to uncover mechanisms controlling trade-offs in locomotor muscle tissue and other equally important organ systems using high throughput sequencing technologies is emphasized by the work on just this single gene. Similar molecular (gene regulatory) mechanisms controlling tissue performance plasticity (i.e. for vision, locomotion, digestion, excretion) likely enable the developmental transition from predatory aquatic larvae to flying adults, and thus could reveal evolutionary signatures important to an aquatic origin of insect flight.

### Thermoregulation and thermal biology

Strenuous activity such as flight tends to raise insect body temperature over ambient due to the relatively low (i.e. 10–20 %) efficiency of muscle power production [115]. As a likely consequence, many flying insect taxa evolved flight muscles that operate optimally at temperatures higher than ambient [166, 174], which in turn necessitated behavioural and physiological mechanisms to tightly control flight muscle temperature. Odonates exhibit a diversity of such thermoregulation strategies and are considered the earliest animal groups to have evolved them, millions of years prior to vertebrates [166, 175].

The odonate thermoregulatory repertoire has been reviewed in detail previously [115, 166]. Percher species typically control body temperature heliothermically, i.e. gaining heat from the sun, through postural adjustments [166, 176, 177] or through shivering thermogenesis (even some gomphids), while other perchers (i.e. some gomphid species, [176]) show little evidence of thermoregulation [178, 179]. Thermoregulation strategies in flier species can additionally involve flight behavioural adjustments (i.e. increase ratio of gliding/powered flight; but see [166]) and/or breathing-assisted blood circulation [180]. The evolution of thermoregulatory strategies in Odonata has likely selected for modifications at the molecular level that optimize and/or stabilize cellular functions at these temperatures. Such molecular thermal sensitivity has been demonstrated in other insect groups (e.g. for lactate dehydrogenase and phosphoglucose isomerase [117, 181]), but has thus far not been examined in Odonata.

In the realm of climatic thermal adaptation, Lancaster et al. [182] used a combination of laboratory experiments and field collections to investigate the genomic responses to sub-optimal temperatures (high and low) in the damselfly *I. elegans*. Thermal chambers were used to expose individuals from central populations and marginal populations in the north of the species’ range to different temperatures, and then RNA-seq was used to examine the number and types of differentially expressed gene transcripts [182]. Several functionally important genes were differentially expressed. Most notably, genes involved in cold tolerance showed a higher evolutionary liability compared to genes associated with heat tolerances. These results will form the basis for future work on climatic adaptation in this and related species. Robust odonate genomic resources would allow us to start examining the molecular consequences of thermoregulatory strategies in this ancient lineage (>300 million years of evolution) and facilitate the integration of thermal biology to better understand the success of Odonata in colonizing environments with vastly different ambient temperatures, and would provide a better understanding of the environmental ecology and dynamics of insects in general.

### Sensory systems

Adult dragonflies and damselflies rely heavily on one sensory system – vision. Although the relative unimportance of smell may have been underemphasized, as recent research has found morphological and electrophysiological data showing that *I. elegans* is capable of detecting the odour of both prey and mates ([183]). Odonata offers an ideal system for studying the evolution of genes involved in vision because visual communication is paramount among Odonata; they have complex colour vision [184], and many behaviours that rely on distinguishing colour [185]. Adults have notably large compound eyes that consist of thousands of ommatidia. The number of ommatidia varies among species from about 7000 in damselflies (e.g. Coenagrionidae) to over 28,000 in large dragonfly species (e.g. Aeshnidae), which are the largest number of ommatidia in any insect eye [186]. Odonates can recognize a wide range of spectra from ultraviolet to red [140, 186, 187], with the light sensitivity differing markedly between the dorsal and ventral portion of compound eyes [139, 188]. Odonates, particularly dragonflies, possess a strikingly large number of opsin genes (light sensitive proteins in the eye that function as the first step in phototransduction) [141], and may have the most complex suite of opsins for any terrestrial animal (e.g. *Anax parthenope* has as many as 30 visual opsin gene copies [141]). These opsin genes are differentially expressed between larvae and adults, and between the dorsal and ventral regions of adult compound eyes [140], which may coincide with the use of different environments between the life stages and sexes. Larvae have smaller eyes that express fewer types of opsins but larger antenna, suggesting that they utilize other cues in addition to vision (e.g. pheromones, vibration or pressure). Indeed, it has been shown that larvae of the damselfly *Enallagma antennatum* rely mainly on chemical cues to detect predators [189].

Accompanying the colour visual system are equally exceptional visual acuity and visual neurons, which make odonates among the most supreme hunters in the animal world, successfully capturing their prey ~95 % of the time [171, 190]. Dragonflies use internal models to regulate sensorimotor control while hunting [170], a trait that was thought to be unique among only vertebrates. Because odonates appear to use mostly one sensory system to carry out relatively complex activities at the adult life stage (e.g. hunting, mate finding), they make an attractive system for the study of vision since these activities are modulated to a large degree by vision alone, i.e. without the contributions and thus the complications of other sensory systems. Furthermore, we know comparatively little concerning the visual system of the larval stage [191–193]. Genomic resources for Odonata will be central for elucidating the diverse potentially co-evolutionary patterns between visual capability, hunting strategy, colour discrimination, colour and colour patterns that occur in the adult and larvae of this group.

### Stress physiology in larvae and integrative physiology

Work on damselfly larvae has been instrumental in exploring physiological stress responses, and has contributed to advancing our understanding of how physiological stress is affected by predation risk, environmental contaminants and responses to combinations of stressors. There is increasing concern that interactions among stressors may negatively affect biodiversity [194]. Specific attention should be paid to understanding how the effects of contaminants may be magnified in the presence of other stressors such as predation risk [195] and higher temperatures [196]. This may explain why contaminant levels assumed to be safe by legislation still cause considerable loss of aquatic biodiversity [197, 198]. To understand and predict synergistic interactions between stressors we need to know how individual and combined stressors affect organismal performance at the physiological level. Yet, even for common natural stressors, such as predation risk, this is poorly known, especially in invertebrates [199]. With regard to physiological effects of predation risk, damselfly larvae are among the best studied invertebrates, which led to novel insights in how predation risk affects prey physiology [200, 201] and thereby can magnify the effects of pesticides [202]. Stress physiology can also help to mechanistically explain how effects of stressors encountered during the larval stage may bridge metamorphosis and shape adult fitness components. For example, it was recently shown that larval exposure to UV stress in the damselfly *C. puella* impairs adult immune function through increased allocation of melanin to the cuticle [203], thereby identifying a novel pathway by which effects of larval stressors can be carried over to the adult stage in animals with a CLC.

Physiological studies are complicated by the often complex interactions between traits that are typically studied in isolation. The development of genomic resources for odonates would allow more rigorous testing of interactions between a changing environment (e.g. temperature, chemical composition) and the abilities of different species to handle environmental stressors. For example, genomic studies could delineate mechanistically the trade-offs between immunity and energetics (e.g. [126]), or more definitively link environmental stressors to impairments in colour development, flight (e.g. thermoregulation ability, flight motor design) and fitness (e.g. [204–206]). The contrast of their quite homogeneous larval life history and ecological relevance (i.e. one of the apex invertebrate carnivores), and highly variable adult form (size, colour, behaviours, physiology, longevity and distribution), combined with broadly based scientific community and rapidly increasing genomics community, identifies Odonata as an model taxon with high potential to achieve such evolutionary and ecologically relevant integration.

## Conclusions

Further development of genomic resources for Odonata could strongly improve research on microevolution driven by anthropogenic environmental changes. Integrating genomic data with the extensive field ecology knowledge of many species could be a major leap forward in the field of eco-evolutionary dynamics [207]. Phenotypic change can come about by adaptation, plasticity or an interaction of the two [207]. Disentangling contributions from these effects is important, as they are expected to be associated with different patterns, rates, limits and costs [207]. Moreover, population genomics could allow the prediction of hybridisation rates and improve the precision of demographic inferences by using dragonflies and damselflies as bioindicator species. This would allow us to plan conservation efforts best suited for Odonata itself, other co-occurring species and their environment. Transcriptomic analyses would allow the identification of genes and molecular processes likely to respond to selection due to climate change and habitat loss (which can be studied across a complex life cycle in Odonata), as recently done by Lancaster et al. [182]. Additionally, reduced representation sequencing approaches for genotyping (e.g. RAD, ddRAD, GBS) make it possible to develop and sequence many markers in non-model species [208], e.g. by sequencing large pools of individuals [209], and hence allow for the detection of outlier loci under selection. Such transcriptomic and genomic studies would benefit from the availability of reference transcriptomes and genomes so that annotation of differentially expressed genes and outlier loci is possible.

### Summary points

1. Odonates constitute an exceptional group to bridge the gap between evolutionary ecology and genomics due to their phylogenetic position, extensive phenotypic and ecological diversity, complex life cycle, ease of study in the wild and usefulness as bioindicators of pollution and climate change.
2. These qualities have made them brilliant study subjects in evolution, ecology and physiology. However, despite the extensive scientific literature, there remains a gap between the availability of genomic tools for Odonata compared to other insect groups (i.e. Holometabola), which prevents the research community from filling the holes in our understanding of insect evolution specifically and arthropod evolution more generally. Closing this gap will lead to insights into some of the most ancient and successful animals on the planet, the insects.
3. Here, we have reviewed and discussed in detail those areas of research where dragonflies and damselflies have provided unsurpassed models to address biologically challenging questions. We have presented a path forward in terms of research and resources needed to connect genomics and evolutionary ecology of this insect group.

### Future prospects

1. Development of key high throughput resources for Odonata, including high-quality genome assemblies and species transcriptomes for both sexes, different tissues and varied ontogenetic life stages.
2. Applying the genomic insights gained from odonates to insects in general, to help elucidate the genomic origins of several evolutionary innovations (e.g. flight).
3. Combining the large ecological dataset available for many species with these resources to analyse macroevolutionary patterns. For example, such a genomics-informed approach would allow us to investigate the widespread colour polymorphisms across the many damselfly species to dissect the genomic basis of colour genes, as well as connect these to the ecological contexts driving colour evolution.

**Box 1. Platform for bioinformatics and genomic resources**

**Table:** 

Genomic research on dragonflies is lagging behind other taxa. By creating a platform where genomic and/or transcriptomic data can be brought together and shared, the available information could be used to its maximum in studies within and across species in this group; allowing us to better understand the evolutionary history of this fascinating and ancient lineage, as well as providing resources for studies of other species across the diversity of insects. The generation of such a platform for dragonflies would facilitate macroevolutionary comparisons of the genome across related species to understand the evolution of genome structure and the phylogenetic relationships of species. Moreover, transcriptomic analyses will be crucial to identify genes and molecular processes involved in adaptation and selection, and in conjunction with genomic data, they could be used to investigate the evolution of gene expression, duplication and function. High-throughput sequencing data may also help to better understand epigenetic changes and genotype-by-environment interactions [18] as well as microevolutionary perturbations (as described in the main text). Furthermore, such data would allow researchers to investigate the large differences in genome size and relate them to biologically meaningful adaptations. Despite the recent advance of high-throughput sequencing technologies the number of these omics resources for dragonflies is yet very limited and scarce. As for May 2016, out of the 261 insect complete genomes available in the NCBI genome database (http://www.ncbi.nlm.nih.gov/genome), only one corresponds to the draft genome assembly of a dragonfly, *Ladona fulva* (BioProject PRJNA194433, Table 1), obtained under the umbrella of the i5k project (http://arthropodgenomes.org/wiki/i5k). Within this same project, two other odonate species are included as “nominated” to have their genomes sequenced: *Libellula depressa* and *Ischnura elegans*. The *I. elegans* draft genome currently has a N50 contig and N50 scaffold size of 4 kb and 39 kb (without gaps), respectively, and a 20 kb library is planned to be added to improve the scaffolding in the near future (Wellenreuther et al. in preparation). The first exploration of the transcriptome in an odonate was done by Simon et al. (2009) who generated 4217 Expressed Sequence Tags (ESTs) for *I. elegans*. The advent of high-throughput sequencing technologies (mainly 454 pyrosequencing and Illumina) has allowed scientists to obtain a large amount of RNA-seq data and to assemble complete transcriptomes for many organisms, but still there is a major contrast in the number of datasets available for odonates when compared to other insects. As of May 2016, a search in the NCBI SRA database (http://www.ncbi.nlm.nih.gov/sra), filtered by RNA data, returned a total of 17,956 datasets for insects, and only 80 corresponded to species within the Odonata. These datasets represent a total of 22 species (Table 1). Additionally, RNA-seq data have been reported for an additional 10 species (e.g. [210, 211]), although these data are not yet publicly available in the NCBI databases. Last, loci for further phylogenomic reconstruction will be extracted from 108 odonate species and these data are expected to be available at the end of 2016 (Karen Meusemann, personal communication). Mitochondrial genomes constitute, to date, the majority of the available complete genomic resources for Odonata, with a total of 14 species, belonging to 9 families; for which complete mitochondrial genomes are currently available (Table 1). Whole mitochondrial genome sequencing allows the study of comparative and evolutionary genomic questions, such as the frequency and type of gene rearrangements and the evolution of genome size, and the integration of nuclear and mitochondrial genome datasets will also help to improve the resolution of future phylogenomic studies [212].	

## Abbreviations

CLC: Complex life cycle
Mya: Million years ago
N/Kg: Newton/Kilogram
PCR: Polymerase chain reaction
SNP: Single-nucleotide polymorphism
SSD: Sexual size dimorphism
